# Can prompts improve self-explaining an online video lecture? Yes, but do not disturb!

**DOI:** 10.1186/s41239-023-00383-9

**Published:** 2023-03-10

**Authors:** Markus H. Hefter, Veit Kubik, Kirsten Berthold

**Affiliations:** 1 Department of Psychology, Bielefeld University, Universitätsstr. 25, 33615 Bielefeld, Germany; 2 Present Address: University of Würzburg, Würzburg, Germany

**Keywords:** Prompts, Online lectures, Video, Self-explaining

## Abstract

In recent years, COVID-19 policy measures massively affected university teaching. Seeking an effective and viable way to transform their lecture material into asynchronous online settings, many lecturers relied on prerecorded video lectures. Whereas researchers in fact recommend implementing prompts to ensure students process those video lectures sufficiently, open questions about the types of prompts and role of students’ engagement remain. We thus conducted an online field experiment with teacher students at a German university (*N* = 124; 73 female, 49 male). According to the randomly assigned experimental conditions, the online video lecture on topic *Cognitive Apprenticeship* was supplemented by (A) notes prompts (*n* = 31), (B) principle-based self-explanation prompts (*n* = 36), (C) elaboration-based self-explanation prompts (*n* = 29), and (D) both principle- and elaboration-based self-explanation prompts (*n* = 28). We found that the lecture fostered learning outcomes about its content regardless of the type of prompt. The type of prompt did induce different types of self-explanations, but had no significant effect on learning outcomes. What indeed positively and significantly affected learning outcomes were the students’ self-explanation quality and their persistence (i.e., actual participation in a delayed posttest). Finally, the self-reported number of perceived interruptions negatively affected learning outcomes. Our findings thus provide ecologically valid empirical support for how fruitful it is for students to engage themselves in self-explaining and to avoid interruptions when learning from asynchronous online video lectures.

## Introduction

The last couple of years, COVID-19 policy measures also affected university teaching and led to a boom in asynchronous online learning (e.g., Guo, 2020; Koh & Daniel, 2022; Lowenthal et al., 2020). Because of such measures, university lecturers had to rather quickly transition their courses into an online format (Schreiber, 2022). One method of choice for many lecturers was to rely on prerecorded video lectures (e.g., Pilkington & Hanif, 2021; Sokolová et al., 2022; Trifon et al., 2021; van der Keylen et al., 2020). This format usually incorporates filming PowerPoint slides and implementing a voice-over recording by the lecturers (sometimes with the lecturer visible in a small box next to the slides). The video file is then distributed by the university’s learning platform and is ready to be digested by students in an asynchronous online learning setting. This asynchronous online learning brings the potential advantages of allowing learners to work through the video lecture at a time and location of their choice. However, it also carries risks, such as interruptions and lack of learning engagement.

Hence, there is a need for measures to encourage students to process such video lectures sufficiently, and many thereof have been researched, such as live tutorial sessions (e.g., Pilkington & Hanif, 2021), or online reflection tasks (e.g., Geraniou & Crisan, 2019). In this paper, we focus on a pragmatic and simple method: enhancing a prerecorded video lecture with adjunct questions, which we call prompts throughout this paper. More specifically, for the current study we analyzed, whether and how a video lecture can actually be enhanced by implementing prompts that should encourage students to deeply process the material. Unlike previous research however, we used an unsupervised asynchronous online learning scenario. Furthermore, we investigate the role of the students’ lack of engagement, such as indicated by the self-reported number of perceived interruptions, on learning outcomes. Our aim was to use an actual authentic university lecture in the field to ensure an optimum of ecological validity.

### The risks of asynchronous video lectures: interruptions and lack of engagement

Asynchronous video lectures enable learners to choose their time and location of learning, but also carry some risks, such as interruptions and lack of students’ engagement. First, the risks of getting interrupted by other people, devices, or noises may be bigger in an unsupervised place of one’s own choice than in the university hall or classroom (e.g., Benson, 1988; Blasiman et al., 2018; Chhetri, 2020). After all, the lecturer’s presence might have at least some corrective influence to assure a quiet learning environment. However, interruptions by digital devices are a ubiquitous phenomenon that afflicts many learners, gaining notoriety under the phrase *digital distraction* (Flanigan & Titsworth, 2020; McCoy, 2020) and the euphemism *media multitasking* (Hwang et al., 2014; May & Elder, 2018). Students simply cannot withstand succumbing to use their mobile devices (such as smart phones, tablets, and laptops) for off-task purposes, which they do to an excessive extent. Already 10 years ago, Burak’s (2012) surveys revealed that over 50% of students engaging in texting—while in fact sitting in class. In online courses, around 70% admitted to texting. Since then, many researchers have contributed various and current (objective) data about students’ digital off-task behavior while sitting in class. For instance, Kim et. al. (2019) found that first-year college students spent over 25% of the time operating their smartphones: every 3–4 min, their smartphone distracted them for over a minute. Moreover, texting students often send and receive between 15 and 20 text messages (Dietz & Henrich, 2014; Pettijohn et al., 2015) during a given class period. Finally, laptop users operate their devices 40–60% of the time for off-task reasons (Ravizza et al., 2017). With such a high amount of digital distraction and off-task behavior—while actually sitting in class supervised by a lecturer (!)—those numbers are presumably even higher while sitting at home supposedly following an asynchronous online lecture. Unfortunately but obviously, such behavior is detrimental to the learners’ academic capacities (e.g., May & Elder, 2018).

Second, there is the risk of lack of students’ engagement, especially in reference to prerecorded video lectures (e.g., Kuznekoff, 2020). There are various reasons for this phenomenon (e.g., Lange et al., 2022). A plausible explanation is offered by Erickson et. al. (2020), who argue that video lectures encourage a more passive learning situation than face-to-face learning in class. However, it is well established that learners’ mental activity is key for learning—not so much their behavioral activity (such as visible open learning activities), let alone mental passivity. This tenet is condensed in the *Active Processing Stance* (Renkl, 2015; Renkl & Atkinson, 2007). Many other researchers argue likewise. For instance, Chi and Wylie (2014) introduced four different modes of learners’ engagement in their *ICAP* framework: *I*nteractive, *C*onstructive, *A*ctive, and *P*assive. They emphasize the benefits of learning activities that go beyond the passive reception of a video lecture that is “watching the video without doing anything else” (p. 221) besides active manipulating the video, such as via play-/pause-buttons. According to this framework, learners can benefit from constructive learning activities such as explaining the concepts seen in the video. Fiorella and Mayer (2016) present similar arguments for the efficacy of encouraging constructive cognitive processing. They discuss the benefits of generative learning strategies in light of their *SOI* framework, which lays emphasis on the cognitive processes *S*electing, *O*rganizing, and *I*ntegrating.

Summing up, for an asynchronous video lecture, it is essential to ensure that learners mentally engage with the learning material and perform a constructive learning activity that extends beyond passive watching. In particular, in this study, we focus on a certain well established and exhaustively researched constructive learning activity, namely self-explaining. Yet the risks of interruptions must not be ignored. Previous research also identified detrimental effects of interruptions on learning activities, such as self-explaining (e.g., Hefter, 2021).

### Prompts for self-explanations

As mentioned above, we are focusing on self-explaining as the key learning activity to encourage students to benefit from a given video lecture. Note that *self-explaining* is not the present participle that describes self-explanatory learning material. *Self-explaining* is the gerund; it is an ongoing activity that simply means generating an explanation to oneself. Decades of research revealed that the endeavor of self-explaining is a powerful learning strategy, a constructive cognitive endeavor (e.g., Bichler et al., 2022; Bisra et al., 2018; Lachner et al., 2021). It means mentally engaging with the learning content, generating inferences, and connecting it with prior knowledge (Chi, 2021; Wylie & Chi, 2014). Usually, students are encouraged by prompts to produce those self-explanations (e.g., Hefter et al., 2022b; Roelle & Renkl, 2020). Many studies have identified the quality with which students generated those self-explanations as an essential predictor for learning outcomes. Not only in an immediate posttest (Berthold et al., 2009; Hefter, 2021) but also in delayed posttests (Hefter et al., 2014, 2022a), self-explanation quality has mediated the effect of digital interventions on learning outcomes.

Hence, it is very plausible that promoting self-explanations is a highly recommended measure to enhance asynchronous online learning (Schreiber, 2022). However, there is still the legitimate question as to how instructors should ideally enhance their video lectures to promote such beneficial self-explanations. More specifically, does being given the simple opportunity to take notes already suffice to promote self-explanations? Do students need to be explicitly induced instead, such as via a prompt? If so, which kind of prompt? Bisra et. al. (2018) argued that an opportunity to generate a spontaneous self-explanation might be more effective than a prompt, because it is more adapted to learners’ individual knowledge gaps. On the other hand, many studies revealed that learners seldom spontaneously engage in self-explaining and do need prompts (or training) to do so (e.g., Berthold & Renkl, 2010). Prompts can also help learners focus on the learning material’s central concepts and principles (*Focused Processing Stance*, e.g., Berthold & Renkl, 2010). Furthermore, the kind of prompt remains an open question to an instructor who wants to enhance a video lecture. There are many different ways to categorize the various types of potential prompts.

In the context of learning from examples or models, a typical differentiation is between principle-based and content-based prompts (e.g., Hefter et al., 2014; Schworm & Renkl, 2007). The principle-based prompts are usually called *learning-domain prompts* and focus on the to-be-learned principles and concepts that underlie the presented learning material (e.g., argumentation principles). By contrast, content-based prompts, usually called *exemplifying-domain prompts*, focus on the part of the learning material that exemplifies the principles (e.g., a discussion about the dinosaurs’ extinction exemplifies argumentation principles). For learning outcomes related to the actual concepts, principle-based prompts have revealed advantages over content-based prompts (Hefter et al., 2014; Schworm & Renkl, 2007).

In the context of learning from explanations, active and constructive prompts can be distinguished (e.g., Roelle et al., 2015). This differentiation is based on the active–constructive–interactive framework by Chi (2009)—a predecessor of the aforementioned ICAP framework (Chi & Wylie, 2014). Active prompts, called *engaging prompts*, should encourage “learners to actively think about the content of instructional explanations” (Roelle et al., 2015, p. 3). By contrast, constructive prompts, called *inference prompts* and tested in combination with reduced explanations should encourage learners to generate something new that goes beyond the originally presented information. However, as Roelle et. al. (2015) aptly discuss, such a constructive endeavor might not only encourage beneficial inferences—it can also lead to learners failing to generate correct inferences. Furthermore, from a more practical point of view, instructors have to think and decide about important aspects, such as whether and how to reduce the number of explanations in their learning material to provide opportunity for inferences, or whether and how to implement additional support measures to deal with potential errors, such as remedial explanations, revision prompts, feedback, or adaption.

Concisely, based on these considerations, we pragmatically focus on two types of prompts, which should be effective for learning from prerecorded online lectures and can also be implemented without the need to alter previous learning material or to implement additional support measures: principle-based prompts and elaboration-based prompts. Principle-based prompts should encourage the learner to think and write about the principles and concepts to be learned. Elaboration-based prompts may be considered similar to the content-based prompts (from the example-based learning content) and the inference prompts (from the explanation-based content). However, they should not require any alteration of the learning material, and simply encourage the learner to think and write about an example situation in which such principles can be applied.

### Hypotheses

In the present study, we supplemented a prerecorded video lecture on the topic *Cognitive Apprenticeship* with different kinds of prompts and compared those types in an online field experiment. Against the previously discussed background, we aimed to investigate the instructional efficacy of different types of prompts (i.e., note prompts, principle-based self-explanation prompts, elaboration-based self-explanation prompts, and both) on students’ learning processes and learning outcomes. Furthermore, we examined the roles of learners’ engagement in the form of interruptions (i.e., self-reports on how often they were interrupted by other people or events/incidents) and persistence (i.e., actual participation in a delayed posttest).

Referring to learning processes and as a manipulation check, we predicted that…

- Principle-based self-explanation prompts would foster principle-based self-explanations (*Hypothesis 1a*).
- Elaboration-based self-explanation prompts would foster elaboration-based self-explanations (*Hypothesis 1b*).
- Combined principle- and elaboration-based self-explanation prompts would foster both principle-based and elaboration-based self-explanations (*Hypothesis 1c*).

Referring to learning outcomes, we predicted that…

- The lecture would foster learning outcomes regardless of the type of prompt (within-subjects comparison; *Hypothesis 2*).
- Principle-based self-explanation prompts would foster learning outcomes (between-subjects comparison; *Hypothesis 3*).

Referring to learners’ engagement during online learning, we predicted that…

- Self-explanation quality would contribute positively to learning outcomes (*Hypothesis 4a*).
- Interruptions would contribute negatively to learning outcomes (*Hypothesis 4b*).
- Persistence would contribute positively to learning outcomes (*Hypothesis 4c*).

## Method

### Sample and design

Three-hundred and seventeen teacher students at a German university participated in the online lecture. Hence, we have learning process data on *N* = 317. Out of these 317 participants, 124 agreed to take part in the posttest immediately after the lecture. Therefore, our main sample that included data on learning outcomes comprised *N* = 124 (73 females, 49 males; *M*_*age*_ = 21.74 years, *SD* = 3.61). Random assignments to the experimental conditions were: (A) note prompts (notes condition, *n* = 31), (B) principle-based self-explanation prompts (principles condition, *n* = 36), (C) elaboration-based self-explanation prompts (elaborations condition, *n* = 29), and (D) both principle and elaboration-based prompts (combined condition, *n* = 28). Out of our main sample of 124 participants, 95 took part in the delayed posttest. These dropouts resulted in varying degrees of freedom (*df*) in the respective statistical analyses. Please see Table 1 for an overview on the conditions and number of participants.

**Table 1:** Procedure, conditions (prompt types), and number of participants

Procedure	Prompt type				Overall
	Notes	Principles	Elaborations	Combined	
Online lecture with prompts	67	91	91	68	317
Immediate posttest	31	36	29	28	127
Delayed posttest after 3 weeks	25	32	19	19	95

### Procedure and materials

This study took place completely online during the summer and winter semesters of 2021/2022. The video lecture took place in the 9th week of the semester, and participants had 3 weeks to access the online lecture on our online platform via their own device’ web browser. After receiving the data protection information and providing informed consent, participants took the pretest on *declarative knowledge* and watched the lectures’ video clips.

This lecture was identical for all our four experimental conditions and featured the topic *Cognitive Apprenticeship* (Collins et al., 1989; Minshew et al., 2021). It was video-based and lasted roughly 40 min in total, showing the last author lecturing and presenting slides. We cut the lecture into six video clips. The first clip was an introduction of about 25 min. Then came four shorter clips lasting about 2 min each that focused on the four main lecture principles. These principles were the components of the *Cognitive Apprenticeship* the students should learn, namely (a) Modelling, (b) Scaffolding and Fading, (c) Articulation and Reflection, and (d) Exploration. The lecture ended with a short outro clip of about 5 min. After each of the four clips about the main principles, a prompt according to the experimental condition was shown. See Table 2 for conditions and prompts.

**Table 2:** Conditions and prompt types

Condition	Prompts
Notes	“Here you can take notes”
Principles	“Describe two central principles of component X”
Elaborations	“Describe how you would implement component X”
Combined	“Describe two central principles of component X” “Describe how you would implement component X”

“X” is a placeholder for the respective component (a) modelling, (b) scaffolding and fading, (c) articulation and reflection, or (d) exploration

After the mandatory video lecture ended, the voluntary part of the study began. This part consisted of the immediate posttest on *declarative knowledge* and *conceptual knowledge* as well as the questionnaire on *interruptions* and demographics, such as age and sex. If participants agreed to take also the delayed posttest on declarative and conceptual knowledge 3 weeks later, we thanked them with 15 Euros.

### Measures

#### Learning time

The online platform we used for the video lecture logged the time that participants spent viewing the four video clips and answering the prompts. The participants could take as much time as they wanted to answer the prompts. The time the participants spent watching the video clips was fixed, though, and the prompts only ever showed up, once the video had finished. Hence, learning time can actually be considered as the sole “prompt-answering” time.

#### Declarative knowledge

To assess learning outcomes, we focused on declarative (i.e., more surface-related) and conceptual (i.e., more depth-related) knowledge. More specifically, declarative knowledge related to a short test comprising eight closed true-or-false items about the lecture’s main principles (i.e., about the Cognitive Apprenticeship Approach). Students could answer them with “true”, “false”, or “do not know.” Scoring for each item was one point for a right answer, minus one point for a wrong answer, and zero points for “do not know.” We summed up the score for all eight items to arrive at a total score on declarative knowledge. We carried out this test three times: right before the lecture (pretest), right after the lecture (immediate posttest), and 3 weeks later (delayed posttest).

#### Conceptual knowledge

To assess deeper conceptual knowledge about the lecture’s main principles (i.e., about the Cognitive Apprenticeship Approach), we posed an open question: “Please describe the main principles of the Cognitive Apprenticeship components.” We rated participants’ answers on a scale from 0 (*minimum*) to 8 (*maximum*), giving up to two points for describing the principles of each the four components. Hence, to receive the maximum rating of eight all four components (i.e., “Modelling”, “Scaffolding & Fading”, “Articulation & Reflection”, and “Exploration”) needed to be correctly described. We assessed conceptual knowledge right after the lecture (immediate posttest) and 3 weeks later (delayed posttest). The first author and a student research assistant were blind to the conditions and rated the data from 25 randomly selected participants (i.e., ~ 20% of the sample). Thanks to high interrater reliability between the two raters (ICC = .98), the student research assistant rated the remaining data.

#### Self-explanation quality

We rated the answers the participants typed in following the four prompts on two scales of self-explanation quality from 0 (*minimum*) to 2 (*maximum*): On the principle-based scale, we gave up to two points for describing the principles of the respective component. On the elaboration-based scale, we gave up to two points for describing an implementation of the respective component. Similar to the rating of conceptual knowledge, the first author and a student research assistant (blind to the conditions) rated the data from 65 randomly selected participants (i.e., ~ 20% of the sample). Thanks to high interrater reliability on both scales (ICC = .85 and ICC = .89), the student research assistant rated the remaining data. We aggregated the sum of all four respective ratings to arrive at a score from 0 (*very low*) to 8 (*very high*) on principle-based self-explanation quality (Cronbach’s alpha = .71) and elaboration-based self-explanation quality (Cronbach’s alpha = .74).

#### Number of interruption

We used a single item as in Hefter (2021) with the following phrasing: ‘Were you interrupted by other people or events/incidents during this web-based lecture?’ and a 5-point scale from 0 (*no interruption*) to 4 (*more than three interruptions*) to assess the number of interruptions. There was a positive significant correlation with learning time, *r* = .29, *p* = .001, underscoring its validity.

#### Persistence

To get some sort of measure of our participants’ persistence, we simply looked and coded whether a participant actually took part at the delayed posttest 3 weeks after the lecture. Hence, persistence was a dichotomous variable (1: *took part*; 0: *did not take part*).

## Results

We applied the classic alpha-level of .05 for all tests. For *F* tests, we reported η_p_^2^ as the effect size. Consistent with prior conventions (Cohen, 1988), effect sizes of η_p_^2^ < .06 were qualified as small, η_p_^2^ between .06 and .13 as medium, and η_p_^2^ > .13 as large. We used a-priori contrasts to compare the different conditions according to our specific hypotheses following suggestions by Furr and Rosenthal (2003). For a descriptive and transparent overview, Table 3 provides means and standard deviations for all our measures.

**Table 3:** Means (with standard deviations in parentheses) for all measures

Measure	Prompt type				Overall
	Notes	Principles	Elaborations	Combined	
Declarative knowledge^a^					
Pretest	1.27 (1.79)	1.32 (2.03)	1.56 (2.78)	1.21 (2.11)	1.35 (2.04)
Immediate posttest	5.71 (1.95)	5.61 (2.30)	6.21 (1.74)	5.57 (2.17)	5.77 (2.05)
Delayed posttest	4.04 (2.82)	5.09 (2.72)	5.42 (3.36)	4.32 (2.81)	4.73 (2.90)
Conceptual knowledge^b^					
Immediate posttest	4.29 (1.99)	3.86 (2.37)	4.00 (2.05)	3.57 (2.13)	3.94 (2.14)
Delayed posttest	4.00 (2.00)	3.03 (2.15)	3.37 (1.83)	3.39 (2.15)	3.41 (2.05)
Self-explanation quality^b^					
Principle-based	3.99 (2.37)	6.47 (1.46)	4.39 (1.72)	6.18 (1.50)	5.28 (2.07)
Elaboration-based	0.05 (0.21)	0.24 (0.60)	2.28 (1.60)	2.31 (1.78)	1.23 (1.63)
Interruptions^c^	0.87 (1.18)	1.17 (1.23)	0.86 (0.83)	1.18 (1.10)	1.02 (1.10)
Learning time^d^	24.16 (14.30)	28.06 (20.99)	34.87 (29.93)	46.56 (24.18)	33.16 (24.73)
Persistence^e^	0.81 (0.40)	0.89 (0.32)	0.66 (0.48)	0.68 (0.48)	0.77 (0.43)

^a^Score from − 8 (minimum) to 8 (maximum)

^b^Scale from 1 (*very low quality*) to 8 (*very high quality*)

^c^Scale from 0 (*no interruption*) to 4 (*more than three interruptions*)

^d^Time in minutes

^e^Dichotomous (1: took part in the delayed posttest; 0: did not take part)

### Learning prerequisites

There were no statistically significant differences between experimental groups with respect to prior declarative knowledge, *F*(3, 313) = 0.48, *p* = .699, η_p_^2^ < .01, reported number of interruptions, *F*(3, 120) = 0.79, *p* = .501, η_p_^2^ = .02, or persistence, *F*(3, 120) = 2.21, *p* = .090, η_p_^2^ = .05. As expected, there was a statistically significant effect of prompt type on learning time, *F*(3, 313) = 12.22, *p* < .001, η_p_^2^ = .11 (medium effect). Answering a self-explanation prompt simply takes more time than not answering (Bisra et al., 2018), especially when it is a combined prompt.

### Learning processes

**Fig. 1:**
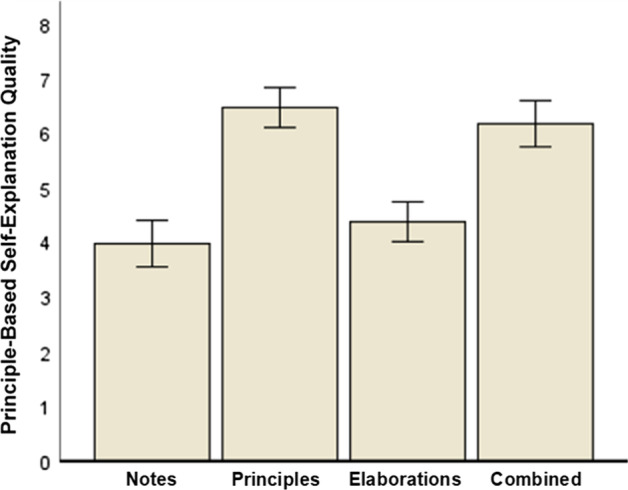
Principle-based self-explanation quality with respect to prompt type. Error bars: 95% CI

First, an ANOVA revealed a large effect of prompt type on principle-based self-explanation quality, *F*(3, 313) = 39.36, *p* < .001, η_p_^2^ = .27 (large effect). Figure 1 displays the results. To test our specific hypotheses that both principle-based (Hypothesis 1a) and combined (Hypothesis 1c) self-explanation prompts foster principle-based self-explanations, we used the following contrast weights assigned to the prompt types: notes: − 1; principles: 1; elaborations: − 1; combined: 1. This contrast test was statistically significant, *F*(1, 313) = 113.43, *p* < .001, η_p_^2^ = .43 (large effect).

**Fig. 2:**
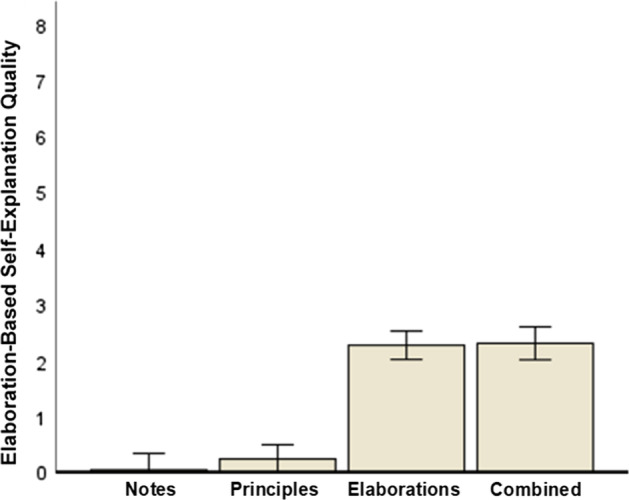
Elaboration-based self-explanation quality with respect to prompt type. Error bars: 95% CI

Likewise, a second ANOVA revealed a large effect of prompt type on elaboration-based self-explanation quality, *F*(3, 313) = 79.19, *p* < 0.001, η_p_^2^ = 0.43 (large effect). Figure 2 displays the results. Again, we used contrast weights to test our specific hypotheses that both elaboration-based (Hypothesis 1b) and combined (Hypothesis 1c) self-explanation prompts foster elaboration-based self-explanations: notes: − 1; principles: − 1; elaborations: 1; combined: 1. This contrast test was statistically significant, *F*(1, 313) = 235.16, *p* < 0.001, η_p_^2^ = 0.43 (large effect).

### Learning outcomes

#### Effect of the video lecture on declarative knowledge

To test our hypothesis that the lecture boosts declarative knowledge regardless of the type of prompt (Hypothesis 2), we conducted two one-way repeated-measure ANOVAs with prompt type as a between-subjects factor, measurement time as a within-subjects factor, and declarative knowledge as dependent variable. The first ANOVA compared pretest and immediate posttest. It revealed a significant effect of measurement time, *F*(1, 120) = 346.52, *p* < .001, η_p_^2^ = .74 (large effect). We found neither a significant effect of prompt type, *F*(3, 120) = 1.50, *p* = .219; η_p_^2^ = .04, nor a significant interaction effect between prompt type and measurement time, *F*(3, 120) = 0.05, *p* = .984, η_p_^2^ < .01.

**Fig. 3:**
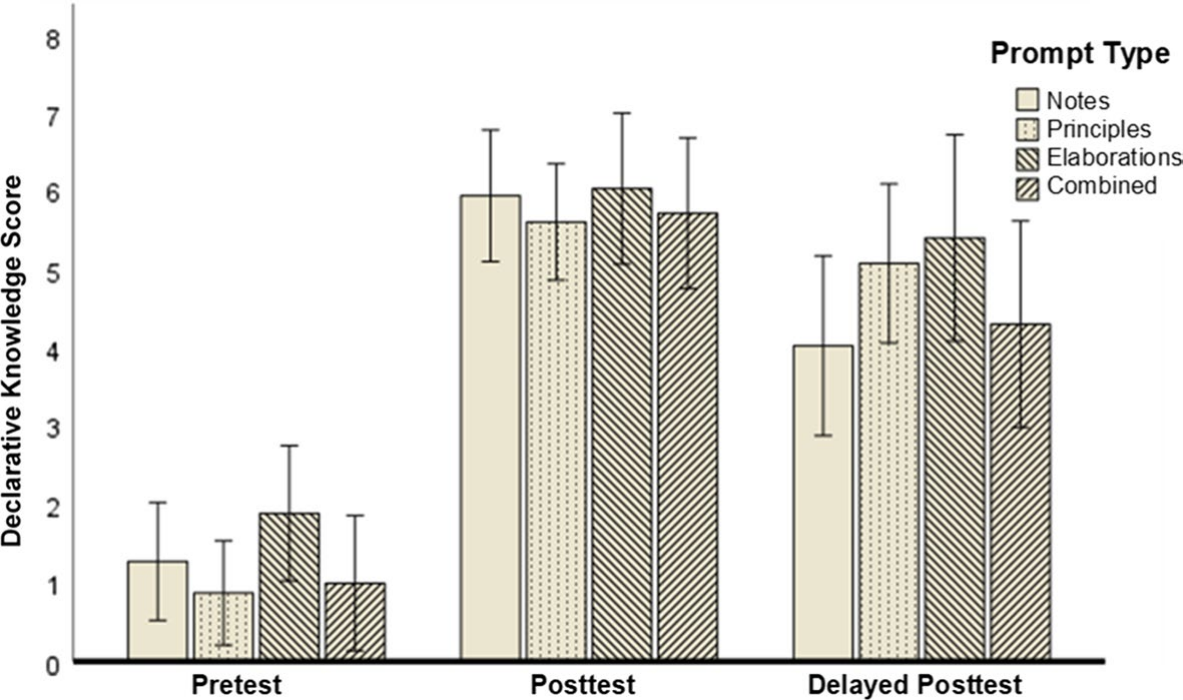
Declarative knowledge with respect to prompt type and measurement time. Error bars: 95% CI

The second ANOVA compared pretest and delayed posttest. Likewise, it revealed a significant effect of measurement time, *F*(1, 91) = 94.79, *p* < .001, η_p_^2^ = .51 (large effect). Again, there was no significant effect of prompt type, *F*(3, 91) = 1.40, *p* = 0.247, η_p_^2^ = 0.04 and no significant interaction effect, *F*(3, 91) = 0.90, *p* = .444, η_p_^2^ = .03. Figure 3 visualizes the declarative knowledge score with respect to prompt type and measurement time.

#### Effects of the prompts on conceptual knowledge

We did not find any significant effect of prompt type on conceptual knowledge (Hypothesis 3) in either the immediate posttest, *F*(3, 120) = 0.57, *p* = .635, η_p_^2^ = .01, or in the delayed posttest *F*(3, 88) = 1.00, *p* = .396, η_p_^2^ = .03. That is to say, we cannot reject the null hypothesis that there is no effect of prompt type on conceptual knowledge.

#### Predictors for conceptual knowledge

**Fig. 4:**
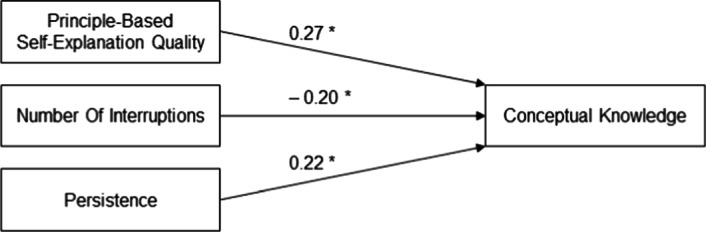
Regression on conceptual knowledge. Standardized beta coefficients, *p < .05

Moreover, we additionally looked at predictors of learning outcomes regardless of prompt type. More specifically, we conducted a multiple linear regression analysis with conceptual knowledge (immediate posttest) as criterion variable and added predictors stepwise. First, we assumed that principle-based self-explanation quality would contribute to learning outcome (Hypothesis 4a). The regression was significant, *F*(1, 123) = 10.60, *p* = .001, *R*^2^ = .08, with principle-based self-explanation quality as a significant positive predictor, β = 0.28, *p* < .001 (one-sided). Next, including the number of interruptions as another an additional predictor (Hypothesis 4b), the regression was significant, *F*(2, 123) = 8.31, *p* < .001, *R*^2^ = .11, revealing an increased amount of explained variance, Δ*R*^2^ = .03. The number of interruptions was a significant negative predictor, β = − 0.20, *p* = .010 (one-sided). Finally, we included persistence as a predictor variable (Hypothesis 4c), resulting in yet another significant regression, *F*(3, 123) = 8.18, *p* < .001, *R*^2^ = .15, and even more explained variance, Δ*R*^2^ = .05. Persistence was a significant positive predictor, β = 0.22, *p* = .005 (one-sided). Figure 4 shows the regression results.

**Fig. 5:**
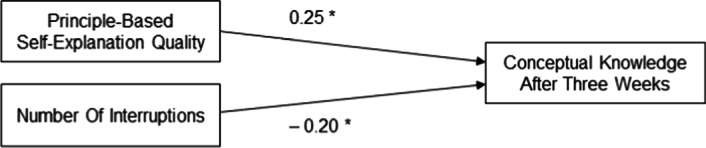
Regression on conceptual knowledge after 3 weeks. Standardized beta coefficients, *p < 0.05

We conducted the same multiple linear regression analysis with conceptual knowledge (delayed posttest) as criterion variable, with the exception that persistence was not included as a predictor variable, because—*per definitionem—*it had the fixed value of 1 for all participants in the delayed posttest. In the first step, we again assumed that principle-based self-explanation quality would contribute to learning outcome (Hypothesis 4a). The regression was significant, *F*(1, 91) = 5.15, *p* = .026, *R*^2^ = .04, with principle-based self-explanation quality as a significant positive predictor, β = 0.23, *p* = .013 (one-sided). In the next step, we included the number of interruptions as an additional predictor (Hypothesis 4b). The regression was significant, *F*(2, 91) = 4.57, *p* =0.013, *R*^2^ = .09, with an increased amount of explained variance, Δ*R*^2^ = .04. The number of interruptions again was a significant negative predictor, β = − 0.20, *p* = .025 (one-sided). Figure 5 displays these regression results.

## Discussion

For the present study, we supplemented a prerecorded video lecture with different kinds of prompts and analyzed learning processes and outcomes. We also aimed to shed some light on the roles of learners’ engagement in the form of number of interruptions and persistence. Relying on an original lecture with actual students in the field had the advantage of maximizing our findings’ ecological validity as they contribute the following to both theory and practice.

### Theoretical contributions and practical implications

First, our findings add to the literature with respect to the effectiveness of different types of self-explanation prompts. We observed the predicted manipulation effects (Hypotheses 1a–c): both principle-based and combined self-explanation prompts fostered principle-based self-explanations. Accordingly, both elaboration-based and combined self-explanation prompts fostered elaboration-based self-explanations. These findings are in line with research about different effects of different prompt types (e.g., Berthold et al., 2009; Roelle et al., 2015; Schworm & Renkl, 2007). Our research adds an important new aspect though, as we achieved our prompt-based benefits on learning processes in an asynchronous and unsupervised online setting. Our results underscore the prompts’ effectiveness in ecological valid learning scenarios, especially when compared to a simple note-taking opportunity that hardly makes students generate self-explanations (cf. Bisra et al., 2018).

Regarding learning outcomes, our within-subjects comparisons revealed that the lecture fostered declarative knowledge regardless of the type of prompt (Hypothesis 2). This result underscores the effectiveness of the lecture, especially concerning a rather surface-related knowledge test, such as answering closed true-or-false items about the lecture.

Our between-subjects comparisons did not reveal any significant effect of prompt type on conceptual knowledge (Hypothesis 3), though. Learners of all conditions performed rather moderately. Hence, for a rather depth-related knowledge test, such as answering an open question about the lecture’s principles, it makes no statistically significant difference, what kind of prompt the learners received. Note that we made between-subjects comparisons referring to conceptual knowledge in the immediate and delayed posttests. Unlike declarative knowledge, there was no pretest on conceptual knowledge for a simple reason: to save our novice learners devoting time and motivation in answering an open question about a topic they probably know nothing about because they still have to watch its lecture.

More interestingly, why did the different prompt types not lead to statistically significant differences in conceptual knowledge? Previous research usually found that—very briefly noted—different prompt types had benefits on certain knowledge types (e.g., Berthold & Renkl, 2009; Schworm & Renkl, 2007). We would like to discuss two potential reasons why this study did not reveal any effects of principle- and elaboration-based prompts on conceptual knowledge. First, we assume that our experimental conditions were similarly effective. After all, learners in all conditions received the identical well-designed video lecture, so that the different types of additional prompts hardly made a difference on conceptual knowledge. As tested and discussed above, our prompts indeed made a difference on self-explanation quality. In particular, the group given principle-based prompts outperformed the group who received simple note prompts concerning principle-based self-explanation quality. However, even this large difference in self-explanation quality might still not be big enough to have an effect on conceptual knowledge. As seen in Fig. 1, the note group still performed reasonably well on self-explanation quality, although they had not received any explicit prompt to do so. However, note that participating in the lecture was mandatory for course credits. Therefore, students in all experimental conditions probably exerted themselves when filling out the textboxes, leading to an equalizing effect between experimental conditions.

Second, the immediate and delayed posttests on conceptual knowledge were part of the voluntary part of the study *after* the mandatory lecture. Hence, the remaining 127 (out of 317, see Table 1) learners were probably more motivated, engaged, and/or interested than their 190 fellow students who decided to opt out. This selection effect might also have equalized the learning outcomes between experimental conditions of prompt type.

To complement our experimental analyses referring to prompt type, we examined potential predictors of learning outcomes. Multiple linear regression analyses revealed (principle-based) self-explanation quality as a positive predictor (Hypothesis 4a) and the number of interruptions (Hypothesis 4b) as a negative predictor for learning outcomes in both immediate and delayed posttests. These findings are in line with previous research. Engaging in high quality principle-based self-explanations is beneficial for learning outcomes (Hefter et al., 2014, 2022a). Furthermore, while learning in an asynchronous online environment, interruptions play a detrimental role (e.g., Hefter, 2021).

From a more practical point of view, these results might be useful for university instructors, because they seem to imply that a lecture is still effective when presented as a prerecorded video in an asynchronous and unsupervised online setting—despite potential diversions and off-task behavior. Our results also advance the idea that instructors should encourage their students to engage cognitively with the learning material, such as by enhancing their video lectures with principle-based self-explanation prompts. These recommendations can be particularly useful for flipped classroom scenarios, which have gained interest for quite some time. Very briefly put, in a flipped classroom scenario, students are provided with online videos to watch at home, whereas the in-class time is spared for interactive group learning activities. Using the flipped classroom approach comes with various requirements and challenges related to IT resources, institutional support, the instructors’ skills, etc. (see Lo & Hew, 2017). Moreover, it is essential that learners deeply process the online video lectures at home, because these serve as the preparation for the learning activities in the upcoming in-class sessions such as discussions, collaborative problem solving, etc. (e.g., Johnston, 2017; Tang et al., 2020). The learners’ deep processing of the video lectures could be supported by implementing principle-based self-explanation prompts. Furthermore, it seems very advisable for instructors to make their students aware of the detrimental effects of interruptions on learning outcomes (e.g., Pattermann et al., 2022)—for instance via short introductory courses about the basics of human learning.

### Future research and limitations

An interesting aspect we noted was the mere participation in the delayed posttest was positively associated with performance in the immediate posttest. One may speculate that this association is based on motivational reasons (such as topic interest), personality reasons (such as conscientiousness), or a mixture of both. Future research might thus further analyze these directions, assess according variables, and test their influence on learning processes and outcomes.

As mentioned above, selection and motivational effects might have equalized the effects of prompt type on the learning outcomes. Many students might have exerted themselves in the mandatory part of study and left afterwards. Future studies might thus rely on non-mandatory video lectures for larger differences between note-takers and self-explainers and less dropout for the voluntary delayed posttest.

Finally, as ecologically valid our asynchronous online setting was, the unsupervised setting brings uncertainty regarding students’ actual behavior when learning with the video lecture. After all, the number of interruptions was a self-report measure, although we have no reason to assume any dishonest responses. For future research, it might be worthwhile to discuss the assessment of more objective log data, such as screen recordings, eye tracking, or even camera recordings—at the cost of resembling a less natural field-like and more unnatural lab-like setting.

Overall, our findings provide ecologically valid empirical support for how fruitful it is for students to engage in self-explaining and to avoid interruptions when learning from an asynchronous online video lecture.

## Data Availability

The data that support the findings of this study are available upon reasonable request.
